# Quench field sensitivity of two-particle correlation in a Hubbard model

**DOI:** 10.1038/srep27189

**Published:** 2016-06-02

**Authors:** X. Z. Zhang, S. Lin, Z. Song

**Affiliations:** 1 School of Physics, Nankai University, Tianjin 300071, China; 2 College of Physics and Materials Science, Tianjin Normal University, Tianjin 300387, China

## Abstract

Short-range interaction can give rise to particle pairing with a short-range correlation, which may be destroyed in the presence of an external field. We study the transition between correlated and uncorrelated particle states in the framework of one- dimensional Hubbard model driven by a field. We show that the long time-scale transfer rate from an initial correlated state to final uncorrelated particle states is sensitive to the quench field strength and exhibits a periodic behavior. This process involves an irreversible energy transfer from the field to particles, leading to a quantum electrothermal effect.

The existence of short-range interaction can induce many exotic physical phenomena, and therefore boost a theoretical interest in a variety of correlated quantum systems. Owing to the rapid advance of experimental techniques, ultracold quantum gases trapped in optical lattices have provided in the past decade a route to simulate the physics of different kinds of correlated quantum systems^1^. For few interacting particles, experiments with ultracold atoms have so far demonstrated the existence of bound pair (BP) states^2^ and correlated tunneling phenomena^3^. This stimulate many experimental and theoretical investigations aiming at the correlated particles, which lead to a series of intriguing novel phenomena involving BP formation^4^,^5^,^6^, detection^7^, dynamics^6^,^8^,^9^,^10^,^11^,^12^, and BP condensate^13^.

On the other hand, recent experiments on optical lattices^14^,^15^,^16^ have renewed the interest for investigating the behavior of correlated quantum system after a sudden change of Hamiltonian parameters, the so-called quantum quench. The corresponding spectacular experimental results have triggered an intensive research on quantum quenches in various systems involving one-dimensional Fermi and Bose systems^17^,^18^,^19^,^20^,^21^,^22^,^23^, Luttinger liquids^24^,^25^ and others^26^. Among many remarkable quench dynamical phenomena, it has been possible to observe collapse and revival of matter waves with Bose-Einstein condensates in optical lattices^27^, coherent quench dynamics of fermionic atoms^28^,^29^, as well as non-thermal behaviors in near-integrable experimental regimes^30^. A straightforward method to realize such quenched systems is the instantaneous change of a global or local parameter of the system like an external field or the interaction strength. In particular, when an external field is applied to the correlated few-body system, preservation of the correlation between the two particles in matter-wave transport can exhibit unusual phenomena that are generally inaccessible in solid-state systems, such as frequency doubling of Bloch oscillation (BO) of two correlated particles^31^,^32^,^33^,^34^, dynamic localization^35^, coherent destruction of tunneling^36^ and the fractional BO of atomic pairs^37^. However, if the external field is applied in a suitable manner, tunnelling between Bloch bands becomes possible, which leads to a sudden death of the BO^38^ or Bloch-Zener oscillation^39^,^40^,^41^,^42^,^43^,^44^.

In previous work^38^, the dynamical behavior of BP has been investigated for the extended Hubbard model driven by an external field. It has been shown that the nearest-neighbor interaction can spoil the completeness of the bound band, which can induce a sudden death of the oscillation of BP and break the correlation between the particles when the external field is switched on. However, the underlying mechanism of such dynamical behaviors is not clear. Motivated by this question, here, we theoretically investigate the underlying mechanism of the dynamics of the two-particle correlation in the framework of the Hubbard model driven by a field. It is shown that the competition between the external field and the short-range interaction can bring about an avoided crossing between the correlated energy level and uncorrelated scattering band leading to the correlation and energy transfer among the involved states. In this case, the initial bound pair will be pumped into the scattering region accompanied with the energy transfer from the field to particles, rather than preserving the correlation of the particles in the unmixed region in which the correlated and uncorrelated states are not mixed. Intuitively, when an external field is applied, there can exist a threshold below which the field cannot induce the destruction of the short-range correlation between the particles. Furthermore, as the magnitude of the field strength is beyond the threshold, the transfer rate from correlated state to uncorrelated states gets larger with the increase of the quench field strength. However, in this paper, we show that the long time-scale transfer rate from an initial correlated state to final uncorrelated particle states exhibits a periodic behavior with the increase of the quench field strength, which can occur in a small range of the field. The essential physics of such counterintuitive dynamics is the periodic occurrence of the field-induced multi avoided crossings between the correlated and uncorrelated energy levels.

## Results

### Model Hamiltonian and the mechanism

For the sake of generality and simplicity, we start from a driven one dimensional extended Hubbard model. It is worth pointing out that the results shown in the following are also applicable to the other form of short-range interaction. The model concerned can be employed to describe the dynamics of interacting particles on a one-dimensional tight-binding lattice driven by an external field, which can be defined by the Hamiltonian


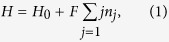


where the second term describes the linear external field served as a quench parameter. And *H*_0_ is the one-dimensional Hamiltonian for the extended Hubbard model on an *N*-site lattice


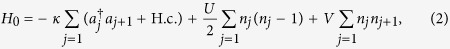


where 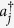 (*a*_*j*_) is the creation (annihilation) operator for boson at site *j* and 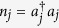 is the number operator. *F* is magnitude of an external field. The tunneling amplitude and on-site interaction strength are denoted by *κ* and *U*, respectively. And *V* accounts for the nearest-neighbor (NN) interaction. The Hamiltonian (2) can also describe ultracold atoms or molecules with magnetic or electric dipole-dipole interactions in optical lattices^45^. In the absence of the external field, the short-range interaction denoted by *U* and *V* can induce the particle pairing with short-range correlation no matter what magnitude of the particle-particle interaction is^38^. We consider a correlated particles state. The spectrum of the system after a field quench is dramatically changed, which may destroy the correlation between the particles. We will demonstrate this point through a simple case.

**Figure 1:**
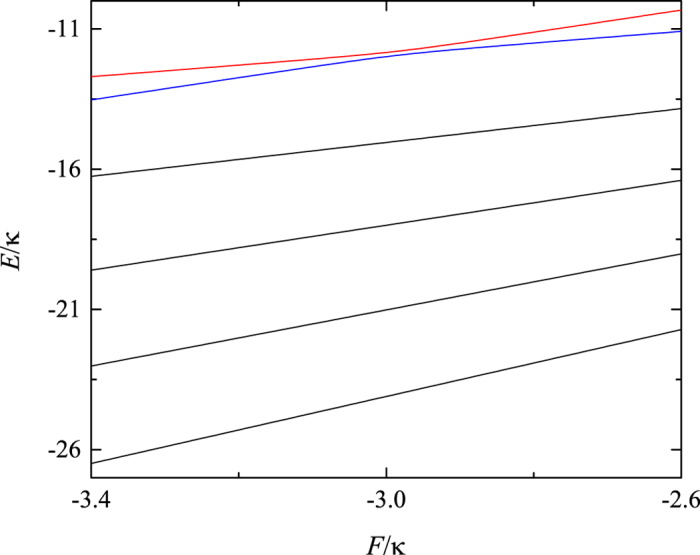
Two-particle energy spectrum of the *N* = 3 system as a function of parameter *F*. The other parameter values of the system are *U* = *V* = −6 and *κ* = 0.4. The avoided crossing involves the top two energy levels denoted by the red and blue line, respectively, which occurs in the vicinity of *F* = *U*/2 = −3.

**Figure 2:**
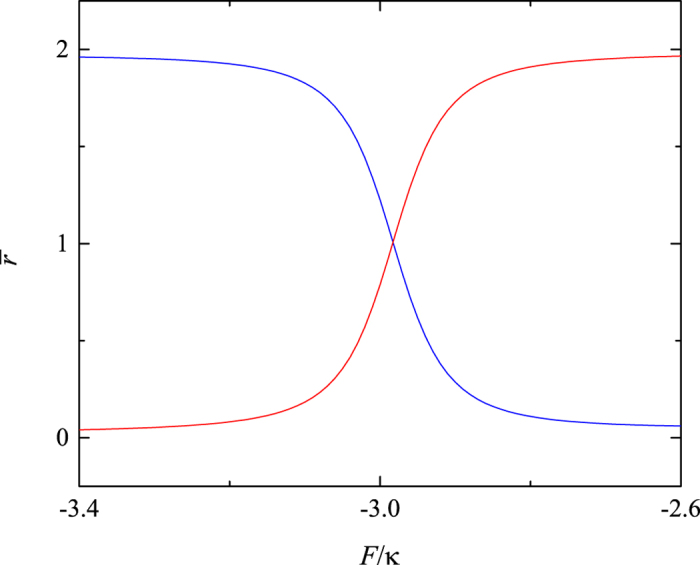
Plot of the relative distance 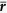 as a function of parameter *F*. The red and blue lines denote the top two energy levels of the system shown in Fig. 1. It can be observed that the two corresponding states exchange the correlation through an avoided crossing region.

We first focus on 3-site chain, which possess a simple energy structure but can reveal the underlying mechanism of the field-induced destruction of the correlation between the particles. The energy levels of the concerned system as a function of *F* with the parameter *U* = *V* = −6 is plotted in Fig. 1. It is shown that there are six energy levels. And we focus on the top two energy levels, which undergo an avoided crossing at the point of *U* = *V* = 2*F*. When the parameters satisfy the condition of 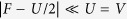 and *F*, 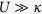, the dynamics of the correlated particles involving top two energy levels is governed by the following effective Hamiltonian


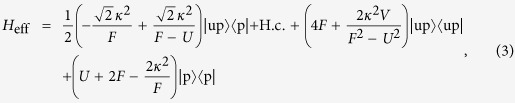


which is shown in detail in Methods section. And 
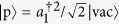
, 
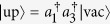
 denote pair and unpair state, respectively. In order to investigate the two-particle correlation of given state, we introduce the average distance between the two particles defined as


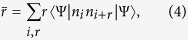


which can measure the correlation between the two particles. We plot the average distance 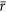 as a function of *F* in Fig. 2, in which the blue and red line denote the correlation of top two energy levels, respectively. It can be seen from that when the external field *F* varies to the vicinity of the point *U* = *V* = 2*F*, the two states are mixed associated with the exchange of correlation through the avoided crossing. Based on this, one can assume that the occurrence of the avoided region can lead the correlated particles to displaying a distinct dynamical behavior for the system with and without avoided crossing. To demonstrate this point, we consider the time evolution of the initial state 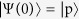, which can be readily obtained as


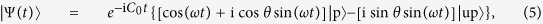


where


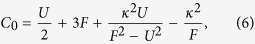



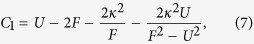



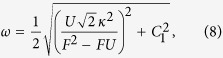



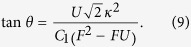


Here we focus on how much probability of the evolved state 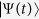 will remain in the state 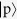 as time goes on. To this aim, we define the transfer rate from the initial paired state to the final unpaired state as


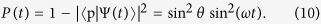


**Figure 3:**
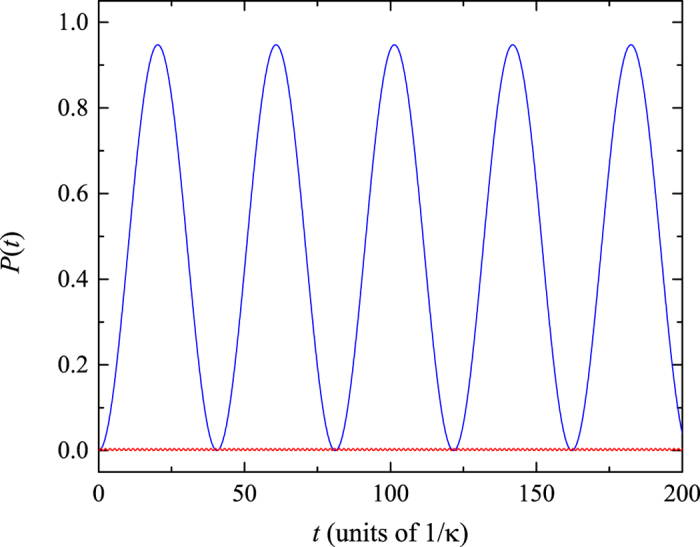
Evolution of the transfer probability *P*(*t*). The other system parameters are the same as that in Fig. 1. The initial state is prepared in the state 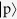. The blue and red lines represent evolutions of *P*(*t*) with the two typical values of *F* = −3, −1, which is corresponding to the system with and without the avoided crossing, respectively.

For the case of *F* = *U*/2 that the parameter is in the avoided crossing region, we have *C*_1_ = −4*κ*^2^/3*U*, *C*_0_ = 4*F* − 8*κ*^2^/3*U*, 
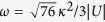
, and 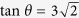, which lead to 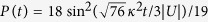. The transfer rate as a function of time is plotted in Fig. 3. It can be shown that the transition between paired and unpaired state occurs with period 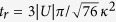, which is inverse proportion to the energy gap between the involved two energy levels. In their essence, the mixing of the pair and unpair state leads to the exchange of their correlation in the region of avoided crossing. On the other hand, when the parameter is away from the avoided crossing region satisfying the condition of 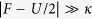, we have *C*_1_ = *U* − 2*F* = 2*ω*, *C*_0_ = *U*/2 + 3*F*, 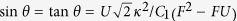, which yield 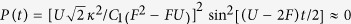 owing to the condition of 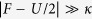. This indicates that the evolved state will remain in the paired state rather than tunneling to the unpaired state as shown in Fig. 3. Through the above analysis, it is clear that the existence of the avoided crossing is crucial for the coherent motion of the correlated particles. However, the structure of the avoided crossing for large *N* system will be something different comparing with that of the above *N* = 3 case. In the following section, we will investigate the large *N* system.

### Energy spectrum and multi avoided crossings

To investigate the influence of an external field on the energy band structure, we first give a two-particle solution of the Hamiltonian *H*_0_. For the sake of simplicity, we restrict the analysis to the system with *U* = *V* < 0, which ensures that the Bloch band for BP locates below the continuum of scattering states. As in refs 12,38, a state in the two-particle Hilbert space can be written as


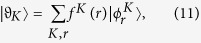


with


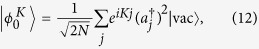



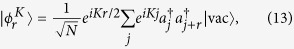


where 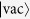 is the vacuum state for the boson operator *a*_*i*_. Here *K* = 2*πn*/*N*, *n*∈1, *N*] denotes the center momentum of two particles, and *r* is the distance between the two particles. Due to the translational symmetry of the present system, we have the matrix representation for the Hamiltonian operator *H*_0_ in the basis 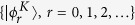



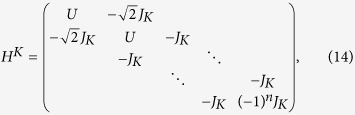


where *J*_*K*_ = 2*κ* = *κ *cos (*K*/2). In its present form, *H*^*K*^ are formally analogous to the tight-binding model describing a single-particle dynamics in a semi-infinite chain with the *K*-dependnet hopping integral *J*_*K*_ in thermodynamic limit *N* → ∞. In this paper, we are interested in the BP states, which corresponds to the bound state solution of the Schrödinger equation


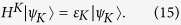


For a given *J*_*K*_, the Hamiltonian *H*^*K*^ possesses one or two types of bound states^38^, which are denoted as 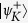 and 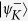, respectively. Here the Bethe-ansatz wave functions have the form of


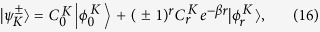


with *β* > 0. For two such bound states 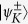 the Schrödinger equation in Eq. (14) admits


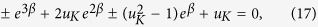


where *u*_*K*_ = *U*/*J*_*K*_ is the reduced interaction strength. The corresponding bound-state energy of 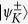 can be expressed as


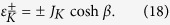


As is shown in ref. 38, if 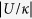 is larger than 6, the system comprises two complete bands, one of which corresponds to two particles bound states. Otherwise, it possesses the incomplete bound band associated with the vanishing of the gap between the scattering band and bound band. Here we want to stress that the preservation of the correlation between the particles in transport does not depend on the completeness of bound band of *H*_0_ as the external field is switched on. The dynamics of the two correlated particles is determined by the existence of the avoided crossings of *H*, which can destroy the pair correlation. We will demonstrate this point in the following section.

**Figure 4:**
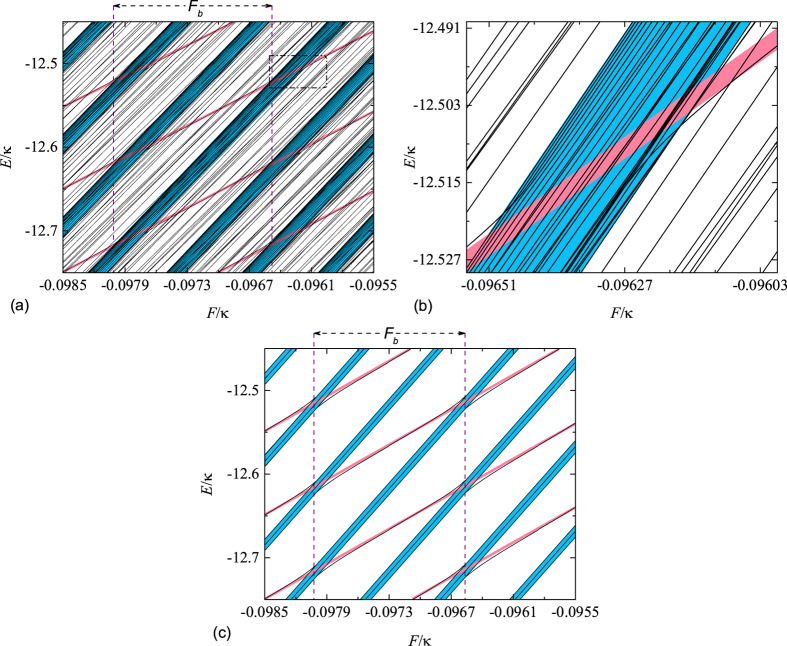
Energy spectrum of the two correlated particles as a function of the external field *F* for the system with *U* = *V* = −6.24, *κ* = 1. The blue and red region indicates correlated energy level and multi uncorrelated energy levels, respectively. It can be observed that a slight variation of the external field can give rise to the periodic occurrence of the avoided crossing region, in which the correlated state and multi uncorrelated states are mixed together. The black dashed rectangle denotes a typical avoided crossing. (**b**) is detail with enlarged scale of black dashed marker in (**a**). (**c**) Schematic illustration of the ideal avoided crossing regions. Each of these contains three scattering and two correlated energy levels. It should be noted that the slopes of the two adjacent correlated lines are slightly different, which is estimated about *F*. This conclusion can also be applied to the adjacent uncorrelated bands composed by multi scattering energy levels.

Now, we switch gears to the spectrum of the Hamiltonian *H*. For the case of 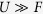, *κ*, the energy spectrum of the two correlated particles becomes discrete and turns out to be composed by two separated Wannier-Stark ladders^44^. In this case, the BP undergoes a perfect BO with period *t*_*B*_ = 2*π*/*F* and preserves the pair correlation, which is analogous to the case of a single particle motion subjected to an external field^34^,^44^. On the other hand, the presence of the external field can not only widen the spectrum of the scattering and bound band of *H*_0_ but also submerge the bound band into the scattering band. In this case, surprisingly, we find that the correlated energy level can overlap with the uncorrelated band formed by multi uncorrelated energy levels. This can be seen from Fig. 4(a), in which the blue correlated energy level overlaps with the first red uncorrelated band, then in turn encounters the different uncorrelated band forming multi avoided regions. In these regions, the corresponding wave functions of two types of energy levels are mixed accompanied with the correlation and energy exchange among the involved states, which can prevent the correlated transport of the two particles. It is worth pointing out that the existence of multi avoided crossing regions is sensitive to the external field *F* and presents a periodic behavior with period *F*_*b*_ = 0.0015 in such a small range of the external field as shown in Fig. 4(a,c). For the sake of clarity and simplicity, we draw schematically the ideal avoided crossing regions between correlated energy level and scattering band and denote the period of the occurrence of these regions in Fig. 4(c). Note that the slope of the two adjacent blue correlated lines are different and so are the red uncorrelated bands, which cannot affect the periodic occurrence of the avoided crossing regions in such a small range of the external field *F*. It is presumable that the dramatic change of the energy spectrum from avoided region to unmixed region induced by a slight variation of *F* can be characterized by the dynamics of correlated particles and exhibits some peculiar behaviors, which will be investigated in the following section.

### Quench dynamics of two-particle correlation

**Figure 5:**
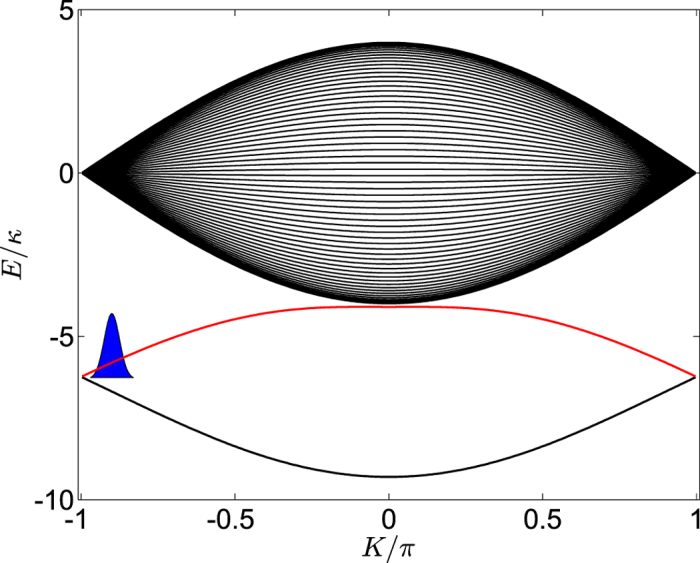
Schematics of the initial correlated wave packet projected in the energy space of *H*_0_. The blue Gaussian wave packet is centered around *K*_0_ = −0.9*π* of the upper bound band denoted by the red line.

To gain insights into the transition of the spectrum induced by the external field. We perform a numerical simulation to investigate the dynamical behaviors of correlated particles. The initial correlated particles state we concerned is placed on the upper bound band of *H*_0_,


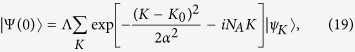


where Λ is the normalization factor, *K*_0_ and *N*_*A*_ denote the central momentum and position of the initial wave packet, respectively. Throughout this paper, we set the parameter *K*_0_ = −0.9*π*, *α* = 0.2, and *N*_*A*_ = 36. For the sake of clarity, we schematically illustrate the initial state 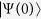 in Fig. 5. We consider a quantum quench in which the external field strength is suddenly changed from *F* = 0 for *t* = 0 to *F* ≠ 0 for *t* > 0. After the quench, the initial state 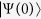 evolves according to the new Hamiltonian 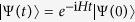. In general, in presence of the external field, one would think that there can exist a threshold beyond which the initial correlated particles state 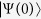 should be pumped into the scattering band and lose their pair correlation. The corresponding transfer rate from the initial correlated particles state to final uncorrelated states gets larger with increase of the field strength. However, after a field quench, the dynamics of the correlated particles state will exhibit a periodic behavior characterized by two distinct behaviors, owing to the dramatic change of the spectrum from the multi avoided crossings to unmixed region in a small range of the external field. This point will be presented in the following.

**Figure 6:**
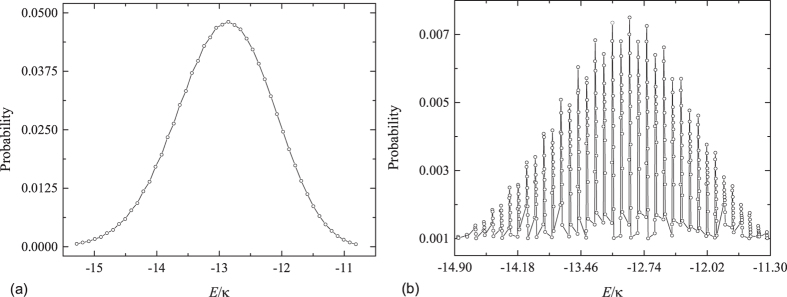
The main distribution of the initial BP wave packet in the energy space for the systems with parameters (**a**) *F* = −0.097120 and (**b**) *F* = −0.097815, respectively. The parameters *U* = *V* = −6.24, *κ* = 1 and *N* = 111 are the same for both systems. For the sake of clarity, we only plot the main distribution of the initial state. 5% of the total distribution are simply not plotted, which refers to more energy levels. And the probability distribution of each of those energy levels is much smaller than that of the energy level plotted in this figure. In (**a**) the profile of energy distribution of the initial state exhibits Gaussian-like form and involves less energy. (**b**) denotes the distribution in the system with multi avoided crossing regions involving more energy levels comparing with (**a**). The interval between the two adjacent energies related to either correlated states or multi scattering states is about *F*, which agrees with our prediction.

Before starting the investigation of the dynamics of the two correlated particles state, we would like to study the distribution of 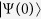 in the energy space for the quenched systems with and without multi avoided crossing regions. The corresponding spectrums of the concerned two systems are plotted in Fig. 4. For the quenched system with multi avoided crossings, the distribution of initial correlated state 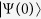 mainly concentrates on the mixed region and therefore refers to many energy levels. This is due to the fact that the correlation exchange takes place among the involved correlated state and uncorrelated band through the avoided crossing. On the contrary, when the sudden change of the field strength *F* is far away from the avoided crossing region, the wave functions of two types of states are not mixed, which suppress the tunneling from the correlated to uncorrelated states. This leads to the fact that the corresponding eigenstates remain their correlation. Therefore, in contrast to the case of the quenched system with multi avoided crossing regions, the initial state 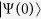 is mainly distributed on the correlated states and contains less energy levels, which are equally spaced with an approximately constant spacing given by *F*. As a comparison, we show in Fig. 6 the main distribution of the initial correlated wave packet 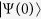 in the energy space of *H* for two typical cases. It can be observed that the energy distribution related to the quenched system with multi avoided crossing regions involves more energy levels comparing with that of the system with the unmixed region, which is in agreement with our analysis. This transition of the distribution can not only reflect the change of the energy spectrum but also result in the entirely different dynamical behaviors of two such quenched systems. Thus it can be presumable that the dynamics of the evolved state 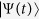 refered to the quenched system without multi avoided crossings will exhibit BO with period *t*_*B*_ = 2*π*/*F* and hold its correlation, which is analogous to a single particle motion subjected to an external field. On the other hand, for the evolved state 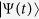 govern by the quenched system with multi avoided crossings, the initial correlated particles will be disassociated and lose the correlation between the two particles through the avoided crossing.

**Figure 7:**
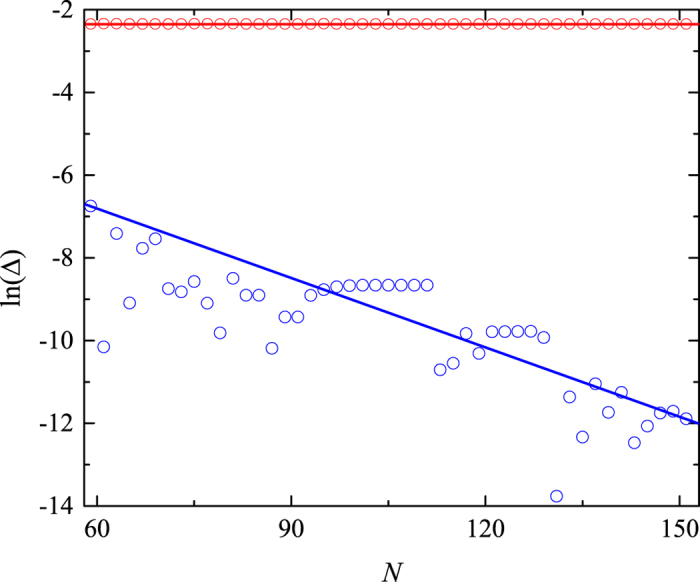
Plot of the logarithm of the commensurability of the involved energy levels as a function of system dimension for the initial state 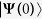. The red circle denotes the corresponding commensurability in the quenched system with field strength *F* = −0.097120. Due to the complexity of the calculation, the blue circles are plotted to represent the up bound of the commensurability in the quenched system with field strength *F* = −0.097815. The other parameters of system are the same with that of the Fig. 6. For the sake of simplicity, the red and blue line are sketched to indicate the tendency of the variation of the commensurability along with the increase of the system size *N*. On can see that, for the initial state mainly distributed on the unavoided energy levels, the commensurability will not change as the increase of the system dimension. On the contrary, for the quenched system with multi avoided crossings, the commensurability is exponential decay with respect to *N*, which indicates that the recovery time of the initial state 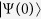 tends to be infinity especially in the the thermodynamic limit and therefore achieves the irreversible process of the disassociation of the initial bound pair.

Here we want to further point out that the dynamics of the disassociation of the two particles is irreversible. Although the system we concerned possesses the time-reversal symmetry, the investigation of the irreversible dynamics in the Hamiltonian with time-reversal symmetry is still of great importance in both the experimental design and theoretical approach. One of the important paradigms is the study of the decoherence in the central-spin model, which has received a lot of attentions in the context of quantum information during the past decade^46^,^47^,^48^,^49^,^50^,^51^. In such a time-reversal symmetric model, the quantum phase transition of the surrounding system can suppress the coherence time of the central spin. However, for the surrounding system with finite spins, the evolution of quantum state will exhibit the periodic behavior with a specific period, which is determined by the commensurability of involved energy levels. The so-called irreversible process is that the recovery time of the initial state is considerably larger than the characteristic time of the system especially in the thermodynamic limit. In our present model, the mechanism of the irreversible process is similar with that of the central-spin model. The bound band and two-body scattering band correspond to the central spin and the surrounding environment, respectively. Comparising with the investigation of the decoherence in the central-spin model, what we focus on is how the quench field can effect on the dynamics of the initial bound pair. For the quenched system with or without avoided crossings, the periodicity of the evolved state depends on the distribution of the initial state in the energy space. From the Fig. 6(a), one can see that the distribution of the initial state is equally spaced for the quenched system without multi avoided crossings, which ensures that the initial bound pair undergoes a Bloch oscillation with periodicity 2*π*/*F*. On the other hand, for the system with multi avoided crossings as shown in Fig. 6(b), the existence of which lowers the commensurability of involved energy levels for the initial state and therefore prolongs the corresponding recovery time. To characterize how the increase of the system dimension can effect on commensurability, we introduce Δ to represent the corresponding commensurability and numerically compute its variation with respect to *N* in Fig. 7. It can be shown that, for the initial state mainly distributed on the unavoided energy levels, the commensurability will not change as the increase of the system dimension. On the contrary, for the quenched system with multi avoided crossings, the commensurability is exponential decay with respect to *N*, which indicates that the recovery time of the initial state 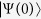 tends to be infinity especially in the thermodynamic limit and therefore achieves the irreversible process of the disassociation of the initial bound pair.

In order to characterize both the above two typical behaviors, as in Eq. (10), we introduce transfer rate 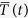 to measure how much probability of the evolved state will remain in the bound states


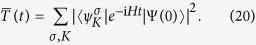


**Figure 8:**
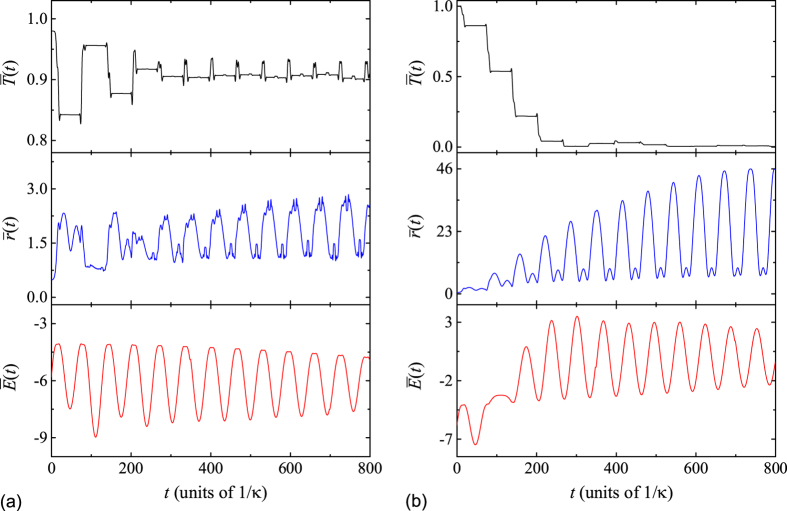
Numerically computed evolution of the functions 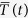, 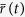, and 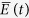 for the initial BP wave packet in the form of Eq. (19) with parameter values given in the text and for two typical values of *F*: (**a**) *F* = −0.097120 and (**b**) *F* = −0.097815. The other parameter values are *U* = *V* = −6.24, *κ* = 1 and *N* = 111. (**a**) shows that the evolved state remains its correlation and preserves the constancy of the average interval energy. This denotes that the two correlated particles undergoes a BO with period *t*_*B*_ ≈ 65. While in (**b**), the increase of 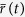, 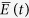 and decrease of 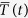 are associated with the Zener tunneling from the bound band to the scattering band, which leads to the dissociation of the two-particle correlation.

We plot Fig. 8 to verify and demonstrate the above analysis. For the evolved state mainly placed on the multi avoided crossing regions, we find that 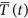 will tend to be 0 accompanied by the irreversible increase of 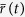 as time goes on. This indicates that the initial two correlated particles lose their correlation, which can be seen from the Fig. 8(b). On the contrary, in Fig. 8(a), the correlation of the two particles remains strong and the corresponding 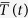 tends to be a steady value 0.93 in a long time scale, which accords with our analytical predictions. Furthermore, the step decrease of 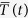 as a function of time in Fig. 8(b) can be explained from another perspective as follows: After a field quench, the presence of the external field can not only drive the correlated wave packet to move along the bound band but also give rise to the Zener tunneling between the bound and scattering band of *H*_0_. The Zener tunnelling takes place almost exclusively when the momentum of the wave packet reaches to the point of center momentum *K* = 0, which is either at the top of the bound band or at the bottom of the scattering band. In this case, the correlated pair can be partially pumped into the upper scattering band accompanied by the destruction of correlation and the increase of energy. At this time, the evolved state consists of correlated and scattering states. Over a period of time, the scattering and correlated components of the evolved state once again arrive at the transition point simultaneously with a characteristic period given by *t*_*B*_ = 2*π*/*F*. Then the two components of the evolved state exchange the energy and correlation from each other due to the Zener tunneling. Through a number of these processes, the 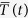 tends to be a steady value. The corresponding time *t*_*f*_ can be termed as quench relaxation time, which deeply depends on the distribution of the initial state in the energy space. To further understand the field-induced energy variation arising from the Zener tunneling from the bound band to scattering band, we study the average energy of *H*_0_, which can be defined by


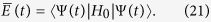


As a comparison, the average energy 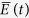 as a function of time for two typical quenched systems is plotted Fig. 8(a,b). In Fig. 8(a), the average energy of the evolved state oscillates around the strength of on-site interaction *U* = −6.24 preserving the constancy of the average interval energy. The corresponding oscillation period is given by *t*_*B*_ = 2*π*/*F*. This implies that the two correlated particles undergo a BO, which is in accordance with our previous analysis. While for the dynamics of correlated particles in the quenched system with multi avoided crossing regions, the average energy of the evolved state is first around the strength of on-site interaction *U* = −6.24 and then oscillates around 0, which indicates that the evolved state is completely pumped into the scattering region with the increase of the average energy of two particles. This can be seen from Fig. 8(b). On the other hand, when the quench field is switched off at time *t*_*f*_, the time evolution of the state 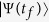 is driven by the Hamiltonian *H*_0_ leading to a constant average energy. At this time, the state 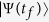 is mainly distributed on the scattering band of *H*_0_ owing to the increase of the average distance 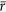, which indicates that the quench process accomplishes the energy transfer from the field to particles. According to the previous discussion, this process is irreversible in the thermodynamic limit. In this point of view, the mechanism of such a transition is analogous to the electrothermal effect in the context of classical physics, which describes the interconversion of thermal energy difference and electrical potential. If we consider the dilute Bose gas formed by many bound pairs in which the pair-pair interaction is neglected, then the mechanism for a single bound pair can be extended to this type of dilute Bose gas. When the quenched field is applied in an appropriate way, the bound pairs are collectively pumped into the scattering region due to the existence of the avoided crossings. Therefore the corresponding temperature is elevated. Based on these, such a dynamical process is irreversible and accomplishes the energy transfer from the field to particles, which suggests a scheme to realize the quantum electrothermal effect.

**Figure 9:**
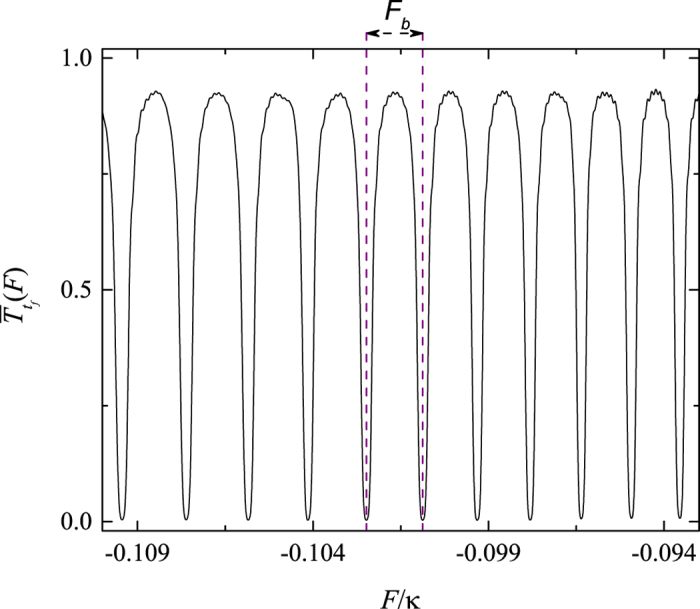
Behavior of the function 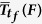 versus the field strength *F* for the parameter *t*_*f*_ = 800. The other parameter values are the same with that of Fig. 4. Note that the 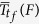 is periodic with period *F*_*B*_ = 0.0015, which accords with the period of periodic occurrence of the multi avoided crossings in the system.

Now we turn to study the sensitivity of the transition between the correlated and uncorrelated particles states after a field quench. In the previous section, we have pointed out that a slight variation of the external field can trigger the periodic occurrence of the multi avoided crossing regions, which can affect the stability of correlated pair. Thus the dynamics of correlated pair is sensitive to the external field and can characterize the change of the energy spectrum. Based on this analysis, we investigate the time evolution of the initial state 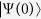 by suddenly quenching the field strength from an initial field-free value to a different value *F*(*t* > 0). And we employ long time-scale transfer rate 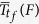 to measure the sensitivity of the correlated-pair dynamics to a quench field, which can be described by


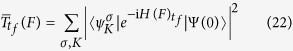


where *t*_*f*_ is the quench relaxation time. At this time, the 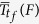 tends to be a steady value. We plot the quantity 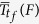 as a function of quench field *F* in Fig. 9. It can be observed that the long time-scale transfer rate 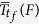 from an initial correlated state to final uncorrelated particle states is sensitive to the quench field strength and exhibits a periodic behavior. And the maximum of 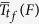 is corresponding to the initial correlated pair placed on the unmixed region of the quenched system. While the minimum of 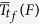 denotes the correlated-pair dynamics in the quenched system with multi avoided crossings. The numerical results clearly show that the 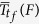 is a good indicator of the transition between systems with and without multi avoided crossing regions induced by the external field. This may also provide a method to identify a subtle change of the system parameter in experiments.

## Discussion

In this work, we have theoretically investigated the coherent motion of two correlated particles driven by an external field in the framework of the one-dimensional Hubbard model. We have shown that the particle-particle interaction can induce BP states, exhibiting short-range quantum correlations. The spectrum comprises two Bloch bands, uncorrelated-particle and BP bands. The presence of the external field can not only changes the energy spectrum from the continuous to discrete but also bring about the mixture among the correlated state and multi uncorrelated states, which is accompanied with the exchange of the energy and correlation between the involved two types of states. We have also shown that a small change of the external field can induce periodic emergence of the multi avoided crossing regions leading to the transition from the initial correlated state to final uncorrelated states. The corresponding transfer rate is sensitive to the quench field strength and exhibits a periodic behavior. This process is irreversible and accomplishes the energy transfer from the field to particles, which could be the mechanism of the quantum electrothermal effect. It is envisaged that the sensitivity of the correlated-pair dynamics to an external field can be applied to detect a subtle variation of system parameter, stimulating further theoretical and experimental investigations on the dynamics of strongly interacting particles in a variety of driven cold-atom systems.

## Methods

In this section, we will show the derivation of the effective Hamiltonian *H*_eff_. We first give the matrix expression for the Hamiltonian (2) in the two-particle invariant subspace.


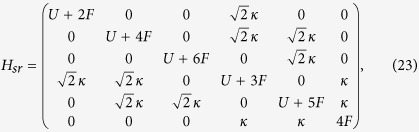


where the corresponding bases are 
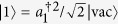
, 
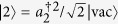
, 
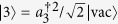
, 
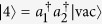
, 
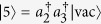
, 
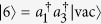
 respectively. What we want to obtain is the effective Hamiltonian describing the two-particle dynamics of avoided crossing region, which is in the vicinity of *F* = *U*/2 = −3 as shown in Fig. 1. When the system parameters satisfy the condition of 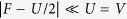 and *F*, 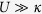, the Hamiltonian *H*_*sr*_ can be divided into following two parts


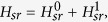


where


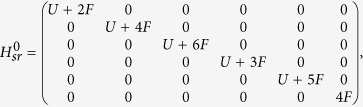


and


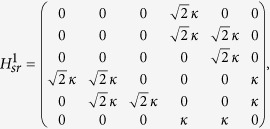


in which the 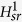 associated with tunneling *κ* can be deemed as the perturbative term. When *κ* = 0, *U* = 2*F*, two-level crossing occurs in which *E*_1_ = *U* + 2*F* and *E*_6_ = 4*F* of the Hamiltonian 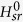 are degenerate. The introduction of the *κ* can change the structure of the corresponding two energies from crossing to avoided crossing. Therefore the eigenstates of the top two energy levels of Fig. 1 are composed by the linear combination of the states 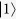 and 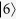. For the sake of simplicity, we denote the two such states by 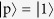 and 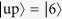. By using the canonical transformation^52^, one can immediately give the entries of the effective Hamiltonian *H*_eff_ referred to the top two energy levels as


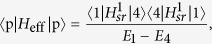



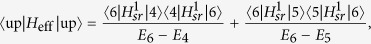



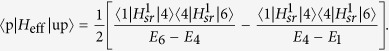


Therefore the effective Hamiltonian can be expressed as


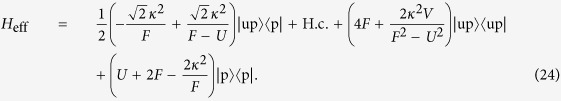


## Additional Information

**How to cite this article**: Zhang, X. Z. *et al.* Quench field sensitivity of two-particle correlation in a Hubbard model. *Sci. Rep.* **6**, 27189; doi: 10.1038/srep27189 (2016).
